# Antioxidation Effect of Graphene Oxide on Silver Nanoparticles and Its Use in Antibacterial Applications

**DOI:** 10.3390/polym15143045

**Published:** 2023-07-14

**Authors:** Hua Jin, Mengyao Cai, Fuquan Deng

**Affiliations:** 1 School of Design and Innovation, Wenzhou Polytechnic, Wenzhou 325000, China; 2 College of Art and Design, Shaanxi University of Science and Technology, Xi’an 710021, China

**Keywords:** GO/AgNPs, antibacterial property, polyacrylate, leather coating

## Abstract

Silver nanoparticles (AgNPs) have drawn great attention due to their outstanding antibacterial effect in a wide range of applications, such as biomass packaging materials, wound dressings, flexible sensors, etc. However, the oxidation of AgNPs limits the antibacterial effect. Firstly, the effects of pretreatment methods on the antibacterial property of AgNPs were investigated by the shake flask method and agar diffusion plate method. Secondly, graphene oxide/silver nanoparticle (GO/AgNPs) nanocomposite prepared by an in-situ growth method was used as antibacterial filler for polyacrylate emulsion via a blending method. The antibacterial mechanism of GO/AgNPs was revealed by comparing the actual contents of oxygen with the theoretically calculated contents of oxygen. Finally, the polyacrylate/graphene oxide/silver nanoparticles (PA/GO/AgNPs) composite emulsion was applied onto a leather surface using a layer-by-layer spraying method to improve the leather’s antibacterial properties. The results showed that ultraviolet irradiation could better maintain the antibacterial property of AgNPs, while GO could improve the dispersibility of AgNPs and prevent their oxidation. The leather finished with the PA/GO/AgNPs-2 wt% composite emulsion showed the highest bacteriostatic rate of 74%, demonstrating its great potential in the application of antibacterial leather products.

## 1. Introduction

Bacterial contamination is a serious threat to human health [1]. However, increasing antibiotic use has resulted in a corresponding increase in microbial resistance, leading to economic and environmental damage, which is a global problem [2]. To solve this problem, antibacterial materials that do not harm human health by themselves have been paid much attention by researchers [3]. Polyacrylate emulsion was widely used in architectural coatings, leather, paper, and other fields due to its high adhesiveness. However, the polyacrylate was colonized by bacteria under the influence of environment during storage and use because of its dense film, so it needs to be imbued with antibacterial properties. Modification of polymers with nanomaterials can give them special functionality [4,5], so the antibacterial property of polyacrylate can be improved by incorporating antibacterial nanomaterials [6].

Silver material or ionic silver possesses outstanding antibacterial effects. For example, using silverware to hold food can reduce the rate of food spoilage, and using gauze made of silver wire to wrap skin wounds can prevent infection [7]. The antibacterial effect of nano silver is much higher than that of macro silver. On the one hand, it can release silver ions; on the other hand, it has a special size effect [8]. The contact between the AgNPs and the cell membrane often causes changes in the structures and properties of the cell membrane [9]. When the particle size of the AgNPs is relatively small (<20 nm), it can interact with the sulfur-containing protein components of the cell membrane, destroy the cell membrane structure and make its permeability increase, then normal function of the cell membrane will be lost until cell death [10,11]. The inherent cytotoxicity of AgNPs affects cells in adverse conditions and produces oxidative stress response, which leads to abnormal accumulation of reactive oxygen species and induces the decay of pathogenic cells, thereby achieving antibacterial purposes in many advanced fields such as biomass packaging materials, wound dressings, flexible sensors, etc. [12,13,14]. Lansdown [15] believes that AgNPs have strong permeability and can penetrate into the subcutaneous tissue, so AgNPs can inhibit the replication of DNA to show a strong antibacterial ability [16].

However, bare AgNPs are prone to form aggregates and be oxidized, so they lose their antibacterial activities during storage and use [17]. Combining AgNPs with other nanomaterials can improve antibacterial action [18,19]. Graphene oxide (GO), as a derivative of graphene, is a new nanomaterial with adjustable size. The monolayer GO surface contains a large number of -OH groups, while there are a lot of -COOH groups at the edge of the layer [20]. These large numbers of oxygen-containing active groups and large specific surface areas make it beneficial to the immobilization and combination of AgNPs [21,22]. At the same time, many researchers believe that the damage of the sharp edges of GO flakes to the cell membrane and the oxidative stress reaction were the major antibacterial mechanism of GO. Therefore, GO and AgNPs were combined to prepare antibacterial nanocomposites that can exert their advantages in more applications [23]. Liu et al. studied the effect of different densities, sizes and shapes of AgNPs on the antibacterial property of GO/AgNPs [24]. The antibacterial activities of GO/AgNPs were found to be twice higher compared to that of AgNPs against both Gram-negative *E. coli* and Gram-positive *B. subtilis* [25]. The amount of AgNPs can also affect the antibacterial activity of the composite [26]. The GO/AgNPs (AgNPs:GO = 1:1) Tang et al. prepared can selectively induce cell death of the *E. coli* and inhibit cell division of Gram-positive *S. aureus*, playing a synergistic antibacterial effect [27]. Zhu et al. considered that the enhanced antibacterial activity of GO/AgNPs was due to the high stability of AgNPs anchored on the GO sheets and the positive-charged surface of hybrids, which increases the electrostatic interaction of bacterial cell membrane with nanohybrids. Cobos et al. [28] thought that AgNPs may be the main contributors to the antibacterial effect of GO/AgNPs. GO/AgNPs sheets exposed at the surface of the film could have a direct contact with the bacteria, while those present in the bulk of the polymer could not. The only possible mechanism of the antibacterial action of the latter was through the release of Ag^+^ ions. Although many studies have shown that the antibacterial properties of GO/AgNPs composites are superior to single GO or AgNPs [29,30], the synergistic antibacterial mechanism of GO/AgNPs composites is still unclear.

In this work, AgNPs, GO, and GO/AgNPs were prepared. Then, their antibacterial properties were tested by the shake flask method and agar diffusion plate method. These two methods were used to understand which kind of presterilization—namely high temperature and UV—is better for AgNPs. The SEM and EDS were used in direct and numerable ways to prove that GO could improve the dispersibility of AgNPs and prevent the oxidization of AgNPs. Moreover, the GO/AgNPs composites were introduced into waterborne polyacrylate emulsion to obtain waterborne PA/GO/AgNPs composite emulsion, which could form antibacterial coatings and could be applied in various fields. Applying the PA/GO/AgNPs emulsion to leather finishing, we also found that using the method of spraying layer-by-layer to increase the contact area between AgNPs and bacteria was more conducive to improving the antibacterial property of leather.

## 2. Materials and Methods

### 2.1. Materials

Silver nitrate (AgNO_3_) was purchased from Shanghai Tri Chemical Reagent Co., Ltd. (Shanghai, China) Sodium citrate (C_6_H_5_Na_3_O_7_•2H_2_O) and dipotassium phosphate (KH_2_PO_4_) were purchased from Tianjin Tianli Chemical Reagent Co., Ltd. (Tianjin, China). Absolute ethanol (CH_3_CH_2_OH) and sodium hydroxide (NaOH) were purchased from Tianjin Hedong Hongyan Reagent Factory. Disodium hydrogen phosphate (NaHPO_4_•12H_2_O) was purchased from Tianjin Kemeiou Chemical Reagent Co., Ltd. (Tianjin, China). Polyacrylate (PA) was purchased from Yan Bang International (Guangzhou) Co., Ltd. (Guangzhou, China) Agar powder, peptone and beef paste were purchased from Beijing Aobaxing Biotechnology Co., Ltd. (Beijing, China).

### 2.2. Preparation of AgNPs

We dissolved 20 mg of AgNO_3_ in 40 mL of deionized water and sonicated for 30 min (45 kHz, 100 W), then moved to a boiling water bath. Then, 20 mg of sodium citrate was quickly added to the aqueous solution and stirred for 30 min. After centrifugation, the AgNPs were obtained.

### 2.3. Preparation of GO/AgNPs Composites

GO was prepared according to the methods in our previous report [31]. We dispersed 10 mg of GO powder in 20 mg of deionized water and sonicated for 30 min in a 45 kHz, 100 W ultrasonic cleaner followed by the addition of silver nitrate solution (20 mg, 50 wt%) and further mixing for 30 min. After transferring to a boiling water bath, 10 mg of sodium citrate was quickly added to the aqueous solution, stirred for 30 min and stood for 6 h, followed by washing with ethanol and deionized water and freeze-drying after filtration to obtain GO/AgNPs composites. GO/AgNPs composites with GO to Ag mass ratios of 1:0, 1:0.3, 1:0.6, 1:1, and 1:1.5, respectively, the samples were recorded as GO/AgNPs-0, GO/AgNPs-0.3, GO/AgNPs-0.6, GO/AgNPs-1, GO/AgNPs-1.5.

### 2.4. Preparation of PA/GO/AgNPs Nanocomposites

Different amounts of GO/AgNPs composites were added to the PA emulsion and blended for 2 h to prepare PA/GO/AgNPs composite emulsion. GO/AgNPs composite materials concentrations in the PA solid content were 0.05%, 0.2%, 0.6%, 1.0%, 2.0%, and 3.0%, respectively. The samples were noted as PA/GO/AgNP-0.05, PA/GO/AgNPs-0.2, PA/GO/AgNPs-0.6, PA/GO/AgNPs-1.0, PA/GO/AgNPs-2.0, PA/GO/AgNPs-3.0. The mixtures were transferred to PTFE molds and dried in the air until the water evaporated completely and formed films. All the films should be kept in a dryer for at least 24 h to minimize the influence of moisture.

### 2.5. Antimicrobial Performance Test

Antimicrobial standard: GB/T 20944.3-2007 Textiles-Evaluation for antibacterial activity-Part 1: Agar diffusion plate method, GB/T 20944.3-2008 Textiles-Evaluation for antibacterial activity-Part 3: Shake flask method. Prior to antimicrobial property tests, AgNPs were sterilized using two methods: (1) ultraviolet irradiation, (2) high temperature treatment to obtain AgNPs-UV and AgNPs-HT.

The antibacterial rate was calculated according to the Formula (1):

(1) $$bacteriost\;atic\;rate(\%)=\frac{A-B}{A}\times 100\%$$

where *A* is average colony number of blank control, *B* is average colony number of antibacterial sample.

### 2.6. Characterization

A Hitachi S4800 scanning electron microscope (SEM, Tokyo, Japan) was used to observe the morphologies of AgNPs and GO/AgNPs. An energy dispersive spectrometer (EDS) was used to observe the oxygen content of GO, AgNPs, and GO/AgNPs.

## 3. Results and Discussion

### 3.1. Characterization of AgNPs

**Figure 1:**
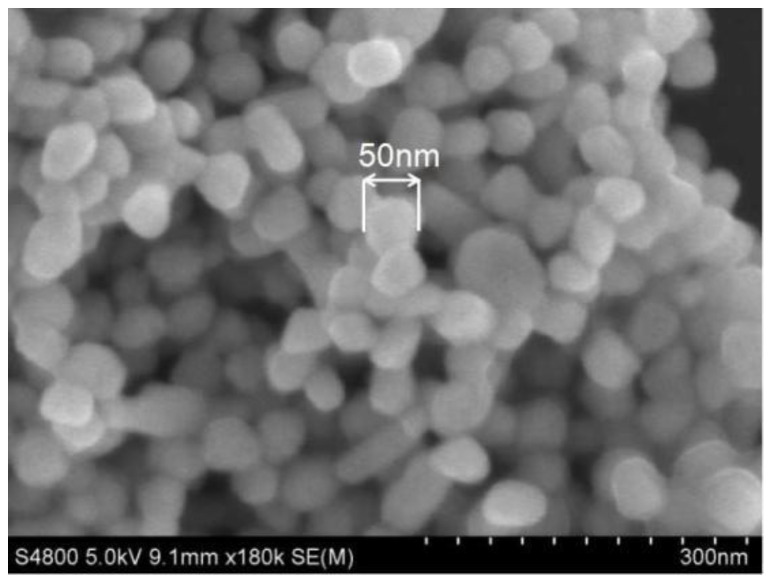
SEM image of AgNPs.

Figure 1 shows the SEM image of AgNPs. AgNPs were prepared by the reduction of AgNO_3_ with sodium citrate. In Figure 1, AgNPs were stacked together with a particle size of about 50 nm. Therefore, AgNPs were successfully prepared [32].

### 3.2. Antibacterial Property

In order to accurately exert their antibacterial properties, antibacterial materials usually need to be sterilized before testing and use in order to avoid contamination; at the same time, the sterilization process should not affect key surface characteristics [33]. There are many different sterilization methods used depending on the desired application and material properties [34]. AgNPs are easy to oxidize and agglomerate at high temperature. To give better play to the antibacterial property of AgNPs, the sterilization method of AgNPs was investigated. Prior to antimicrobial property tests, AgNPs were sterilized using two methods: (1) ultraviolet irradiation, (2) high-temperature treatment to obtain AgNPs-UV and AgNPs-HT.

**Figure 2:**
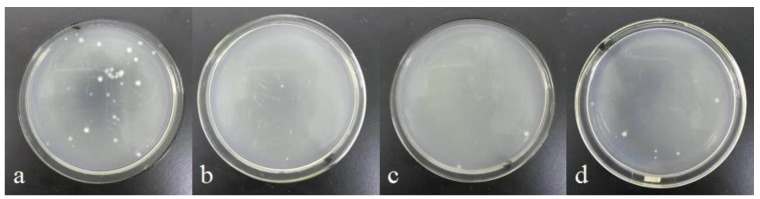
Optical images of the antimicrobial activity of samples by the shake flask method: (**a**) blank, (**b**) AgNPs, (**c**) AgNPs-UV, (**d**) AgNPs-HT.

**Figure 3:**
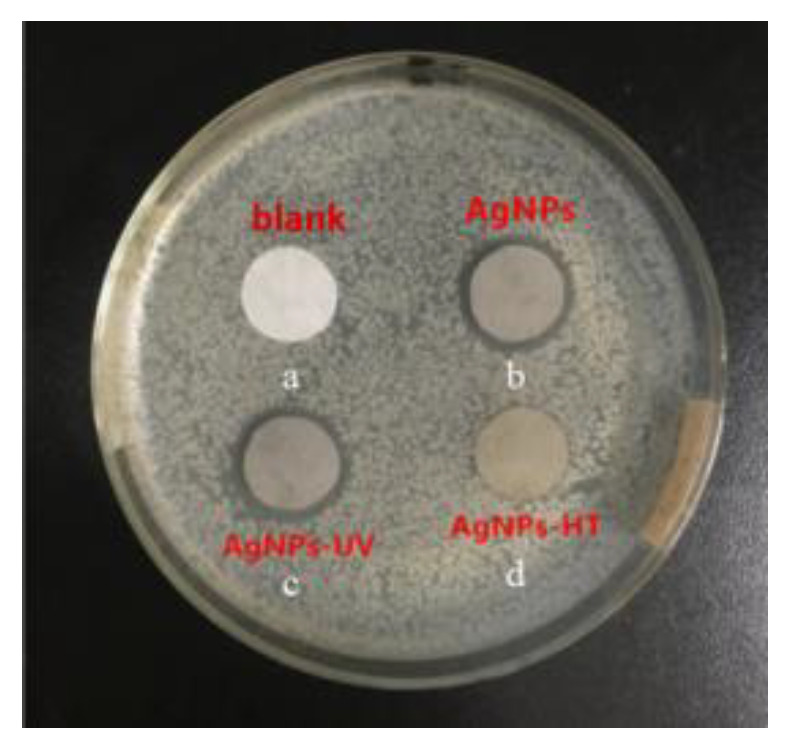
Optical images of the antimicrobial activity of different samples by the agar diffusion plate method: (a) blank, (b) AgNPs, (c) AgNPs-UV, (d) AgNPs-HT.

A blank sample was used as the control group to investigate the effect of different sterilization methods on the antibacterial properties of AgNPs. Figure 2 shows optical images of the antimicrobial activity of different samples by the shake flask method. The number of bacteria colonies in AgNPs medium (Figure 2b) and AgNPs-UV medium (Figure 2c) were greatly reduced compared to the blank control group (Figure 2a), which indicating that both of them have antibacterial activity against Gram-negative *Escherichia coli*. The calculated bacteriostatic rate of AgNPs and AgNPs-UV was 91.66% and 94.44% by the colony counting method, respectively. However, there were a few bacteria colonies in the AgNPs-HT medium (Figure 2d), and the bacteriostatic rate was 75.00%. The same result (Figure 3) was shown in the antibacterial properties test by the agar diffusion plate method. The width of the bacteriostatic circle of AgNPs and AgNPs-UV were both 1.5 mm. The width of the bacteriostatic circle of AgNPs-HT was 0 mm, which meant that the antibacterial property of AgNPs treated by high-temperature were not effective. This could be ascribed to the strong reducibility of AgNPs, which gave them high-efficiency sterilization ability. However, after being treated by the high-temperature sterilization, some AgNPs were oxidized, and Ag^+^ cannot be dissolved out, which results in a decrease in their antibacterial property. At the same time, some studies pointed out that the size and morphology of the AgNPs treated by the high-temperature sterilization may show a dramatic change. Most AgNPs were further aggregated from smaller particles to large agglomerates, which could not perform an effective antibacterial effect [35].

**Figure 4:**
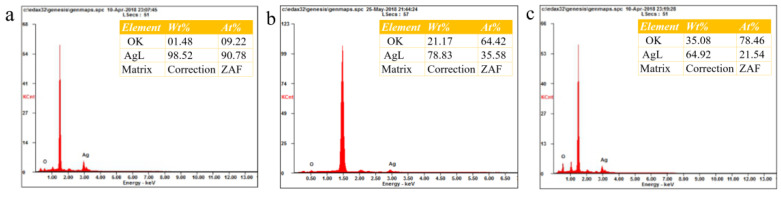
EDS results of AgNPs after different sterilization treatments: (**a**) AgNPs, (**b**) AgNPs-UV, (**c**) AgNPs-HT (inset: element content of different samples).

In order to figure out the cause of the low antibacterial properties of AgNPs-HT, the EDS characterizations of AgNPs, AgNPs-UV, and AgNPs-HT were displayed in Figure 4. The oxygen content of AgNPs was 1.48%, and after treatments with different methods, the oxygen content of AgNPs-HT increases significantly (35.08%) and was higher than that of AgNPs-UV (21.17%), which proves that AgNPs were partially oxidized to form Ag_2_O after being treated by the high-temperature sterilization, which would cover the AgNPs surface. The oxidation level of AgNPs-UV was lower than that of AgNPs-HT, which indicates that UV treatment is a more promising method.

**Figure 5:**
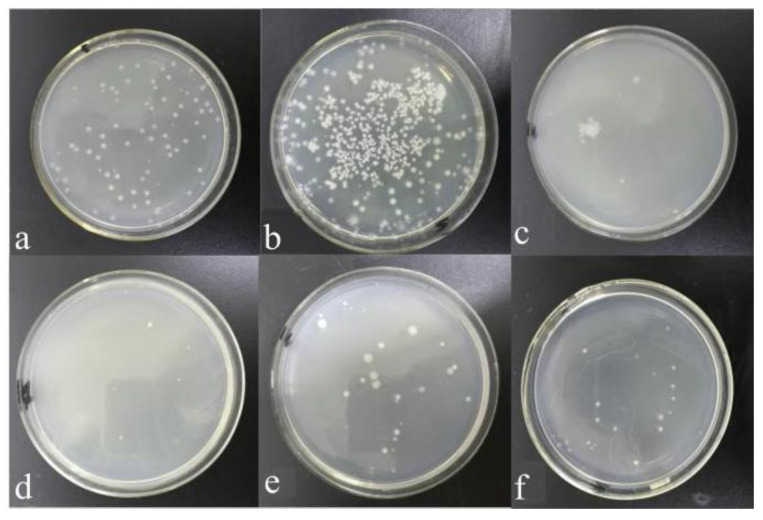
Optical images of the antibacterial activity of GO/AgNPs with different ratios: (**a**) blank, (**b**) GO/AgNPs-0, (**c**) GO/AgNPs-0.3, (**d**) GO/AgNPs-0.6, (**e**) GO/AgNPs-1, (**f**) GO/AgNPs-1.5.

GO was found to possess an antimicrobial effect on the Gram positive bacteria [36]. Therefore, combining GO with AgNPs was expected to obtain a higher antibacterial property than that of single nanomaterials. Different proportions of AgNO_3_ were added to the GO solution, the AgNPs grew on the surface of GO nanosheets to obtain the GO/AgNPs composites. Figure 5 shows the antibacterial property results of GO/AgNPs composites with different mass ratios of GO and AgNPs. Except for the GO/AgNPs-0, the colony number of GO/AgNPs composites with different ratios all decreased, showing good antibacterial properties. As the proportion of AgNPs in GO solution was increased, the bacteriostatic rates tended to increase first and then decrease. When the ratio of GO/AgNPs was 1:0.6, the bacteriostatic rate of the composite material against *E. coli* was the highest at 99.27%. Therefore, the antibacterial property of GO/AgNPs composites were closely related to the composite ratio of GO and AgNPs.

**Figure 6:**
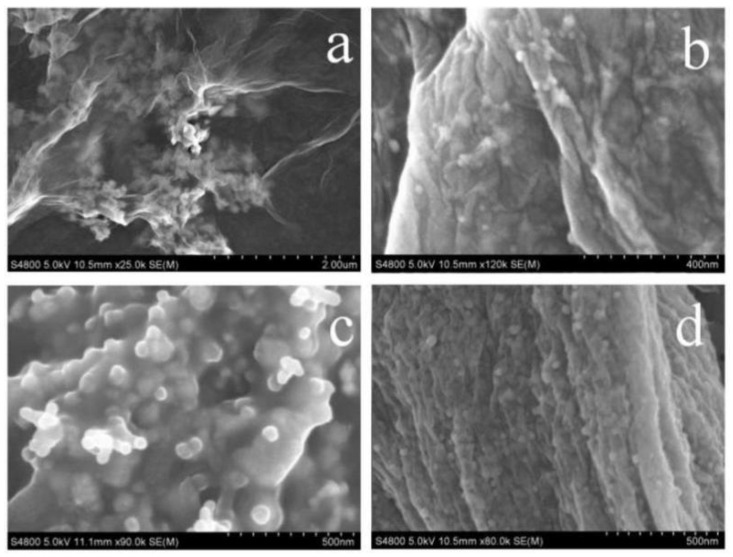
SEM images of GO/AgNPs with different ratios: (**a**) GO/AgNPs-1: 0.3, (**b**) GO/AgNPs-1:0.6, (**c**) GO/AgNPs-1:1, (**d**) GO/AgNPs-1:1.5.

Interestingly, the prepared GO did not show any antibacterial effect (Figure 5b). However, the GO/AgNPs-1:0.6 shows a similar bacteriostatic rate with that of the neat AgNPs, indicating that GO can improve the antibacterial property of AgNPs. In order to explore the reasons for the high antibacterial property of GO/AgNPs-1:0.6, GO/AgNPs with different compounding ratios were characterized by SEM and EDS. Figure 6 displays the SEM images of GO/AgNPs with different mass ratios, where the round nanoparticles were AgNPs, and the wrinkled nanosheets were GO [37]. The size of AgNPs were not changed, at about 50–60 nm, which showed that there was little effect on the growth of AgNPs with the introduction of GO. When the GO: AgNPs ratio was 1:0.3, there were only a small amount of AgNPs, which could only cover a small part of the GO surface (Figure 6a). When the ratios of GO/AgNPs were 1:1 and 1:1.5, the AgNPs aggregated to form large-sized agglomerates (Figure 6c,d), which could not be well dispersed. Only when the GO/AgNPs ratio was 1:0.6 was the AgNPs content moderately and evenly distributed on the surface of GO (Figure 6b). Therefore, GO was beneficial to the dispersion of AgNPs, which could further improve the antibacterial property of AgNPs [38]. On the one hand, when the ratio of GO to AgNPs was too high, the contents of AgNPs in the nanocomposites per unit area were small, which resulted in unsatisfying antibacterial property of the GO/AgNPs. On the other hand, when the composite ratio of GO to AgNPs was too low, the AgNPs could not be well dispersed. It was easy to form large-sized agglomerates, resulting in decreased antibacterial property of the GO/AgNPs. An appropriate ratio of GO to AgNPs could promote the decentralization of AgNPs to improve the antibacterial property of GO/AgNPs.

**Figure 7:**
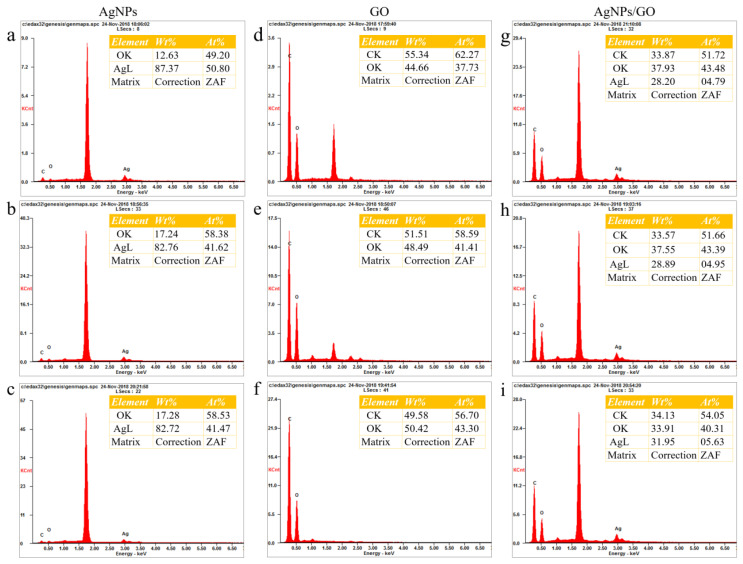
EDS results of AgNPs with different drying time: (**a**) 0 min, (**b**) 15 min, (**c**) 30 min. EDS results of GO with different drying times: (**d**) 0 min, (**e**) 15 min, (**f**) 30 min. EDS results of GO/AgNPs with different drying times: (**f**) 0 min, (**g**) 15 min, (**h**) 30 min (inset: element content of different samples).

Furthermore, GO was dried in an oven for 0 min, 15 min, 30 min at 60 °C, respectively (AgNPs and GO/AgNPs were dried by the same method). EDS analysis was performed on each sample to observe their oxidation degree (Figure 7). It is assumed that all the elemental carbon and silver in GO/AgNPs originated from GO and AgNPs, respectively. Since AgNPs would be oxidized in air, elemental oxygen came from both GO and AgNPs. The content of oxygen derived from GO in GO/AgNPs can be calculated based on the carbon–oxygen ratio in GO, and the content of oxygen derived from AgNPs in GO/AgNPs could be calculated based on the silver–oxygen ratio in AgNPs. Therefore, the theoretical content of oxygen in GO/AgNPs could be calculated according to the Formula (2):

(2) $$O_{theoretical}=C_{3}\cdot O_{1}/C_{1}+Ag_{3}\cdot O_{2}/Ag_{2}$$

where 1, 2, and 3 in the subscript represent GO, AgNPs, and GO/AgNPs. According to Formula (2), the theoretically calculated content of oxygen in GO/AgNPs with drying times of 0 min, 15 min, and 30 min were 31.41%, 37.64%, and 41.38%, respectively. The actual value of the content of oxygen could be obtained from the EDS results of GO/AgNPs with different drying times in Figure 7f–i. Through Formula (3), the difference between the theoretical and actual contents of oxygen in GO/AgNPs nanocomposites could be calculated:

(3) $$\Delta O=O_{actual}-O_{theoretical}$$

**Figure 8:**
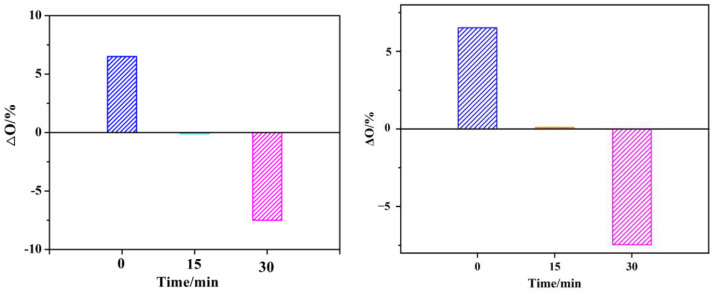
Difference between measured oxygen content and theoretical oxygen content in GO/AgNPs with different drying time.

Figure 8 depicted the difference between the actual and theoretical contents of oxygen in GO/AgNPs with different drying times. The trend of the difference between the actual and theoretical contents of oxygen as drying time increased could be observed. Firstly, the value of actual content of oxygen in GO/AgNPs was 6.52%, which was higher than that of the theoretical value. However, after drying the GO/AgNPs nanocomposites for 15 min, the actual content of oxygen was 0.09% less than the theoretical value. After 30 min of drying, the actual value of content of oxygen was 7.47% less than the theoretical value. As drying time increased, the ΔO decreased and became negative, which indicated that the oxidation degree of AgNPs in GO/AgNPs was decreased. Therefore, the addition of GO played a role in preventing the oxidation of AgNPs [39].

**Figure 9:**
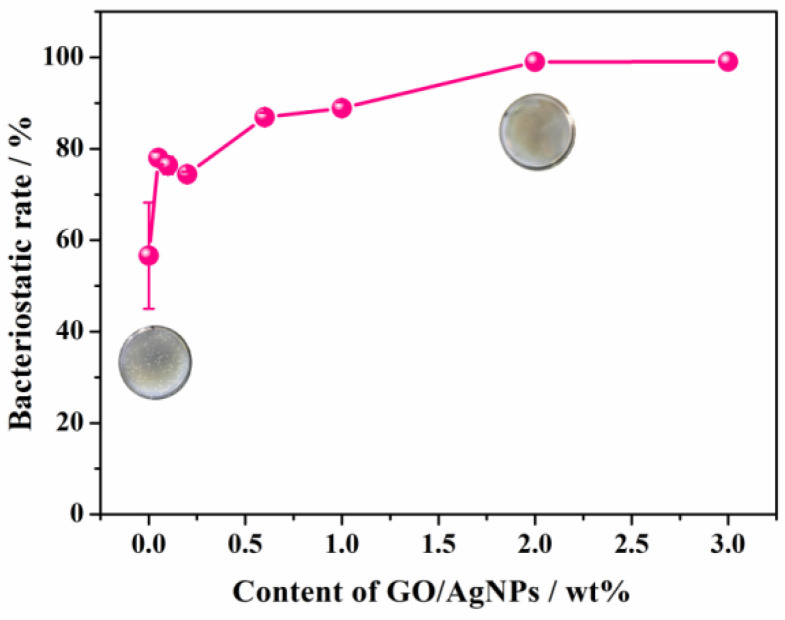
Bacteriostatic rate curve of PA/GO/AgNPs with different content of GO/AgNPs.

In order to prepare the composite emulsion with good antibacterial property, different amounts of GO/AgNPs nanocomposites as filler were incorporated into PA emulsion. According to Formula (1), the bacteriostatic rate of the composite emulsion with different contents of GO/AgNPs was calculated, which is shown in Figure 9. With the increase of contents of GO/AgNPs, the bacteriostatic rates of the PA/GO/AgNPs composite emulsion gradually increased. When the content of the filler was 2%, the growth of bacteria was almost inhibited, and the bacteriostatic rate reached 99.00%. Therefore, the optimal dosage of GO/AgNPs was 2%.

**Figure 10:**
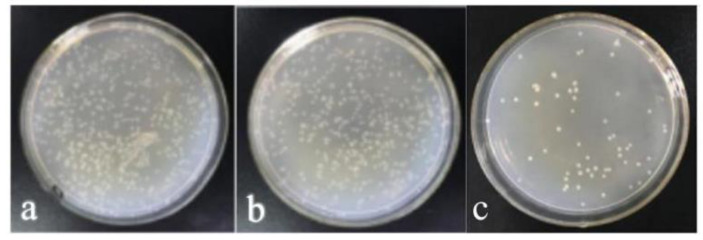
Optical images of the antibacterial activity of the leather samples coated by different spray methods: (**a**) uncoated leather, (**b**) leather sample coated by a normal spraying method, (**c**) leather sample coated by a layer-by-layer spraying method.

**Figure 11:**
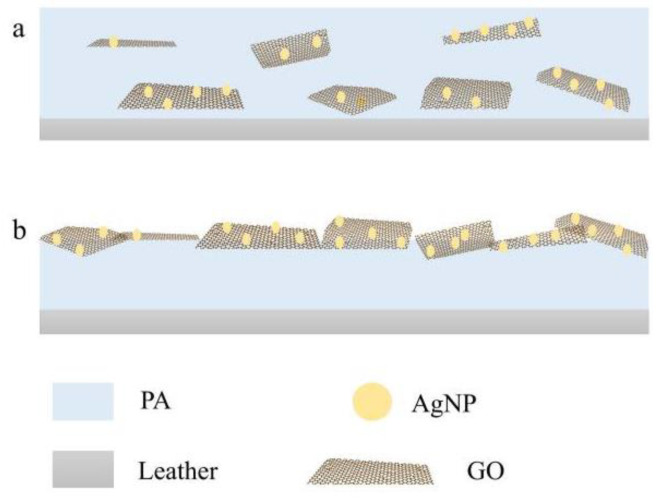
Schematic diagram of the GO/AgNPs distribution on leather samples coated by different spray methods: (**a**) leather sample coated by a normal spraying method, (**b**) leather sample coated by a layer-by-layer spraying method.

PA/GO/AgNPs-2% composite emulsion was sprayed on the leather surface by a normal method to form a coating. Then, the antibacterial property of the coated leather sample was observed. The number of colonies on the coated leather sample was still high (Figure 10b). The bacteriostatic rate was only 42.36%, which was far lower than that of the composite film (99.00%). This may be ascribed to the fact that leather, as a biomass material, is a good surface on which to breed bacteria; at same the time, AgNPs were wrapped by PA emulsion, so the AgNPs were not in contact with the bacteria. In order to improve the antibacterial property of coated leather, PA was first sprayed on the leather surface. After the PA emulsion dried, GO/AgNPs-2% was sprayed on the PA surface to obtain double-layered coated leather using a layer-by-layer method. Obviously, the bacteriostatic rate was dramatically increased to 74% (Figure 10c). Therefore, the layer-by-layer method was more conducive to the improvement of the antibacterial property of the coated leather [40]. Spraying PA emulsion first, and then covering GO/AgNPs, could expose the AgNPs to the surface of the leather (Figure 11b), which made AgNPs easier to contact the bacteria and perform the antibacterial action. However, AgNPs were wrapped by PA in the PA/GO/AgNPs composite film (Figure 11a), so only a small amount of AgNPs could be exposed to air, making it hard for AgNPs to release Ag^+^. So, the antibacterial property was weak.

## 4. Conclusions

In conclusion, AgNPs as prepared possessed remarkable antibacterial properties. The antibacterial property of AgNPs-UV was better than that of AgNPs-HT. The AgNPs were anchored on the surface of GO by an in situ growth method to prepare GO/AgNPs nanocomposites. Although GO did not show an obvious antibacterial effect, GO/AgNPs showed better antibacterial action than AgNPs. The mechanism could be attributed to the fact that GO could improve the dispersibility of AgNPs and prevent their oxidation, as proved by the distribution of AgNPs and the comparison of the oxygen content of the composite materials after heating treatment. Moreover, the PA/GO/AgNPs-2% composite emulsion was sprayed on the leather surface by a layer-by-layer method, which gave the leather good antibacterial properties by increasing the contact area between AgNPs and bacteria, demonstrating its great potential in the application of leather products. This work contributes to the explanation of the mechanisms of the good antibacterial properties of GO/AgNPs nanocomposites.

## Data Availability

The data presented in this study are available on request from the corresponding author.
